# Microbial Analysis of Bite Marks by Sequence Comparison of Streptococcal DNA

**DOI:** 10.1371/journal.pone.0051757

**Published:** 2012-12-19

**Authors:** Darnell M. Kennedy, Jo-Ann L. Stanton, José A. García, Chris Mason, Christy J. Rand, Jules A. Kieser, Geoffrey R. Tompkins

**Affiliations:** 1 Sir John Walsh Research Institute, School of Dentistry, University of Otago, Dunedin, New Zealand; 2 Department of Anatomy, University of Otago, Dunedin, New Zealand; 3 Department of Preventative and Social Medicine, University of Otago, Dunedin, New Zealand; Cairo University, Egypt

## Abstract

Bite mark injuries often feature in violent crimes. Conventional morphometric methods for the forensic analysis of bite marks involve elements of subjective interpretation that threaten the credibility of this field. Human DNA recovered from bite marks has the highest evidentiary value, however recovery can be compromised by salivary components. This study assessed the feasibility of matching bacterial DNA sequences amplified from experimental bite marks to those obtained from the teeth responsible, with the aim of evaluating the capability of three genomic regions of streptococcal DNA to discriminate between participant samples. Bite mark and teeth swabs were collected from 16 participants. Bacterial DNA was extracted to provide the template for PCR primers specific for streptococcal 16S ribosomal RNA (16S rRNA) gene, 16S–23S intergenic spacer (ITS) and RNA polymerase beta subunit (*rpoB*). High throughput sequencing (GS FLX 454), followed by stringent quality filtering, generated reads from bite marks for comparison to those generated from teeth samples. For all three regions, the greatest overlaps of identical reads were between bite mark samples and the corresponding teeth samples. The average proportions of reads identical between bite mark and corresponding teeth samples were 0.31, 0.41 and 0.31, and for non-corresponding samples were 0.11, 0.20 and 0.016, for 16S rRNA, ITS and rpoB, respectively. The probabilities of correctly distinguishing matching and non-matching teeth samples were 0.92 for ITS, 0.99 for 16S rRNA and 1.0 for rpoB. These findings strongly support the tenet that bacterial DNA amplified from bite marks and teeth can provide corroborating information in the identification of assailants.

## Introduction

A bite mark is defined as a physical alteration in a medium caused by contact with the teeth [1]. Bite marks have provided crucial physical and biological evidence for the prosecution of violent crimes [2]. Bite marks can be found in inanimate objects such as foodstuffs, however it is injuries inflicted on human tissue that comprise the majority of bite mark cases presented in court [3]. Human bite marks are sustained predominantly in homicide, sexual assault and child abuse [4].

The examination of bite marks currently relies on morphometric analysis, which involves the comparison of the characteristics of a suspect’s teeth with full-scale photographs of the injury [5]. The correlation of a bite mark to the dentition of a suspect utilizes parameters of size, shape and alignment of teeth in addition to dimensions of the dental arch [5]. The forensic discipline of bite mark analysis is centered on two assumptions; firstly, that the characteristics of the teeth involved in biting are unique to an individual, and secondly, that this asserted uniqueness is registered in the material that is bitten. The term “forensic” means “pertaining to a court of law” [6] thus bite mark evidence has been admissible testimony in criminal proceedings for almost 60 years [7]. Despite the importance placed upon this evidence, there has been rising concern regarding the lack of empirical evidence underpinning conventional bite mark analysis [8], [9], [10], [11], [12], [13], [14]. These concerns were recognised in the National Academy of Sciences report released in 2009 which concluded that “no evidence of an existing scientific basis for identifying an individual to the exclusion of all other” could be found [15].

DNA profiling was developed in the 1980’s and over the last 20 years, the adaptation of this technology permits DNA from human biological sources to be used for identification purposes. In cases involving bite marks, the recovery of human DNA from saliva provides an objective form of evidence [8]. However, nucleases, such as deoxyribonuclease I, present in saliva at relatively high concentrations [16], [17], [18] contribute to the rapid degradation of exposed DNA [19]. Because of the difficulties that can be encountered in recovering salivary DNA of sufficient quality and quantity to generate a DNA profile, an alternative objective approach to bite mark analysis has been directed toward a bacterial genotyping method [20], [21], [22].

Bacterial DNA is enclosed within the cell envelope, which provides a biological barrier against the degradation suffered by exposed human DNA. More than 700 bacterial species inhabit the human oral cavity [23] and the numerically dominant species belong to the genus *Streptococcus* [24], [25], [26]. 16S rRNA gene sequence analyses [27], [28] has shown that oral streptococci are included within four multispecies phylogenetic units: the anginosus, mitis, mutans and salivarius groups. Currently, member species of these phylogenetic units include: *S. anginosus*, *S. constellatus* and *S. intermedius* (anginosus group); *S. australis*, *S. cristatus*, *S. gordonii*, *S. infantis*, *S. mitis*, *S. oralis*, *S. parasanguinis*, *S. peroris*, *S. pneumoniae* and *S. sanguinis* (mitis group); *S. cricetus*, *S. downei*, *S. ferus*, *S. macacae*, *S. mutans*, *S. orisratti*, *S. rattus* and *S. sobrinus* (mutans group); *S. salivarius*, *S. thermophilus* and *S. vestibularis* (salivarius group).

Characterization of the microbiota of the oral cavity reveals that *S. mitis*, *S. oralis* and *S. sanguinis*, are the initial colonizers of the teeth [23], [29], [30], [31]. Of these species, *S. mitis* (which exhibits considerable genotypic diversity) is the predominant organism [30], [31], [32], [33], [34]. Humans harbour multiple strains of the same *Streptococcus* species with many strains seemingly unique to individuals [32], [33], [35]. This intraspecies diversity provides the premise that oral streptococci isolated from a bite mark inflicted on human skin may be genotypically matched, with a high degree of assurance, to those from the teeth responsible [20], [21]. These observations were reiterated in a third study [22] that circumvented the need for prior culturing by amplifying bacterial DNA directly from teeth and experimental bite marks. In that study, streptococcal DNA, amplified with primers specific for hypervariable region 9 of streptococcal 16S rRNA gene, was resolved by denaturing gradient gel electrophoresis (DGGE), and a comparison of the amplicon profiles from the bite marks and teeth matched most bite marks to the teeth responsible. However, there was a concomitant risk of false positives with the sole use of this relatively conserved locus [22].

Phylogenetic analysis and identification of bacterial species have been conventionally based on 16S rRNA gene sequence comparison; however, the variable regions contained within this locus are generally insufficient for distinguishing closely related streptococcal species [22], [36]. Alternative gene targets that discriminate between closely related streptococci include ITS (stretch of non-coding DNA that lies between the 16S and the 23S rRNA genes) [37], [38], [39], [40], *rnpB* (encoding endoribonuclease P) [41], [42] and *rpoB* (encoding the beta subunit of the bacterial RNA polymerase) [43], [44]. The variability offered by these regions is sufficient for discriminating between streptococcal species with almost identical 16S rRNA gene sequences. Therefore, the current study focused on determining whether such variability enables the discrimination of strains. Should these alternative molecular targets facilitate strain differentiation then it may be feasible to utilize them to distinguish between individuals.

This investigation had two objectives: the first was to apply high throughput sequencing, using the GS FLX 454 technology, to assess the feasibility of matching oral streptococcal DNA sequences amplified from experimental bite marks (inflicted on human skin) to those obtained from the teeth responsible. The second was to evaluate the capability of three genomic regions of streptococcal DNA to discriminate between participant samples.

## Materials and Methods

### Ethics Statement

The study design was approved by the University of Otago Human Ethics Committee (January 16, 2008, reference number 06/169). Written consent was obtained from all participants.

### Bite and Teeth Samples

Sixteen unrelated adult participants recruited from the staff and students of the University of Otago generated self-inflicted bites on their upper arms [20], [21], [22]. Participants were healthy adults who had not used mouthwash in the preceding month or antibiotics in the preceding three months. Before inflicting the bites, a sterile cotton applicator moistened in 0.9 % saline, was used to swab the area of skin to be bitten, to provide an index of the bacteria naturally present on the skin and to facilitate the distinction between oral and skin bacterial sequence data. Participants firmly bit their own upper arm in the bicep region with enough force to leave clear impressions of the teeth that would last for at least five minutes. Three hours later, saline-moistened cotton applicators were used to swab the bite mark. Dry, sterile cotton applicators were used to sample the upper and lower anterior teeth at this time also. The tips of the applicators were placed into separate sterile tubes each containing 2 ml of saline, and were vortexed for 30 seconds to detach the bacteria.

### DNA Extraction

Extraction and purification of bacterial DNA from the skin, bite mark and teeth samples was achieved with InstaGene™ matrix (Bio-Rad Laboratories, Hercules, CA) according to manufacturer’s protocol. Portions (1.5 mL) of the saline-suspended bacteria were centrifuged for 3 minutes at 11,000 rpm at 4°C. The supernatant was discarded and the pellet resuspended in 200 µL of InstaGene™ matrix. Preparations were incubated at 56°C for 30 minutes, vortexed for 10 seconds and heated in a boiling water bath for 8 minutes. The tubes were cooled to room temperature, vortexed for 10 seconds and centrifuged for 2.5 minutes at 11,000 rpm at 4°C. An aliquot (100 µL) of the supernatant containing extracted bacterial DNA was recovered and stored at −20°C.

### PCR Amplification

The streptococcus-specific oligonucleotide primers for the amplification of approximately 245 base pair (bp) fragments of the 16S rRNA gene; 16S–23S rRNA intergenic spacer region (ITS); endoribonuclease P (*rnpB*); and RNA polymerase beta-subunit (*rpoB*) loci are given in Table 1. Primers for the 16S rRNA gene and *rnpB* fragments have been previously described [42], [45]. Alignment of partial ITS and *rpoB* sequences, from numerous strains of oral streptococci catalogued in GenBank, (http://www.ncbi.nlm.nih.gov/nuccore) identified areas of high variation and primers were selected in conserved flanking regions. All primers included the GS FLX/454® (Roche) Adapter A (for forward sequencing, GCCTCCCTCGCGCCATCAG) and B (for reverse sequencing, GCCTTGCCAGCCCGCTCAG) fused to the 5′ end of each primer.

**Table 1:** Forward (F) and Reverse (R) primers for the amplification of 16S rRNA (16S), ITS, *rnpB* and *rpoB* fragments for GS-FLX DNA sequencing.

Loci	Primer sequence (5′ to 3′)	Approxiate amplicon length (bp) including primers
16S-F	GAGGTTGATCATGGCTCAG	238–248
16S-R	ACAACGCAGGTCCATCT	
ITS-F	AGGTAGCCGTATCGGAAGGT	231–250
ITS-R	YACAGCGTTTTCGGTTTATTT	
rnpB-F	GTGCAATTTTTGGATAATCG	248–254
rnpB-R	TGGGTTGCTAGCTTGAGG	
rpoB-F	CTGAAGAACGTCTCTTGCAC	245
rpoB-R	CGAGAGACAACCCCTTTRTT	

PCR was performed in simplex with 5 µL of template DNA in a total reaction volume of 50 µL consisting of 37.8 µL of nuclease-free deionised water, 5 µL of 10X Taq buffer (25 mM Tris-HCl [pH 8.0], 35 mM KCl, 2.5 mM MgCl_2_) (HotMaster 5 PRIME GmbH, Hamburg, Germany), 1 µL of deoxyribonucleoside triphosphates (10 mM) (Roche Diagnostics, Indianopolis, USA), 0.5 µL of each primer (0.1 µM) and 0.2 µL of Taq DNA Polymerase (5 U/µL) (HotMaster). Thermocycling was preceded by an initial denaturation at 94 °C for 1 minute with maintenance at 4 °C following the last cycle. Reactions were subjected to 35 cycles (DNA Engine Thermal Cycler, Bio-Rad, CA, USA) of denaturation at 94 °C for 30 seconds, annealing at 56 °C for 30 seconds and extension at 72 °C for 30 seconds. PCR products were purified on silicate columns (QIAquick, Qiagen GmbH, Hilden, Germany) and the concentration of each eluate was estimated visually following agarose gel (1.5%) electrophoresis and staining with ethidium bromide.

### Pyrosequencing

For the first 11 participants the four amplicon libraries were pooled (in equimolar amounts) to give 11 bite mark and 11 teeth samples. For participants 12–16, the amplicon libraries were not pooled. All bite mark and teeth samples were sequenced individually. Samples were loaded into a 16-lane bead deposition gasket on a 70 X 75 mm PicoTiterPlate (Roche). Sequencing was performed in both forward (A-adapter sequence) and reverse (B-adaptor sequence) directions with the standard (not Titanium) amplicon sequencing protocol for the GS FLX/454® (Roche).

### Data Analysis

The filtering pipeline designed to extract high quality reads comprised three levels. The first and third levels were executed using a customized computational pipeline and the second employed an open source workflow. In the first level, reads shorter than 220 bp were discarded and the remaining reads grouped according to their locus. In the second level, the workflow Galaxy (galaxyproject.org) removed both forward and reverse primer sequences and eliminated bases with a PHRED quality score of < 20 (removing ambiguous base calls). Where ambiguous bases occurred, the read would be truncated. Therefore, the third filtering level discarded reads shorter than 180 bp and determined the frequency at which each read was observed. Reads observed only once were discarded. For reads observed at least twice, the script indicated their frequency in the sequence header. However, in this final data set, the read was represented as the consensus read. Thus the data set comprised high quality unique reads only. A minimum of ten unique reads/data set was required for samples to be included in comparative analyses. An additional customized script enabling the direct comparison of bite mark and teeth reads disclosed the number of reads 100% identical between the two sample types.

Prior to this study, a control experiment was performed to determine the quality of the filtered and trimmed reads isolated using this customised workflow. The pipeline processed read data from a sample containing a defined amplicon mix (reference sequences for amplicons were obtained using Sanger sequencing) and an error rate of 0.106% was determined (manuscript in preparation). While the error rate is lower than the 0.25% previously reported for GS FLX platforms [46], a maximized stringency was maintained by including only reads of 100% identity between two sample types. The proportion of shared identical reads was calculated by dividing the number of identical reads shared between a bite mark sample and a teeth sample by the total number of reads in that bite mark sample. For 16S rRNA, ITS and *rpoB*, all shared identical reads were compared with sequences available in the nucleotide database of GenBank (http://blast.ncbi.nlm.nih.gov/Blast.cgi) to identify SLOTUs (species-level operational taxonomic units).

### Statistical Analysis

Statistical modelling provided estimates of parameters for a population based on the sample data. Logistic regression is the preferred model for analyzing binary outcome variables. The statistical parameters generated from this analysis determined: i) whether a relationship existed between the binary outcome variable and the predictor variable and; ii) the optimum proportion of shared identical reads yielding the greatest probability of correctly matching a bite mark to the corresponding teeth. Statistical analyses were undertaken with R (http://cran.r-project.org/).

The 16S rRNA, ITS and *rpoB* read data from each bite mark and teeth sample were compared to determine the proportion of shared identical reads between the two sample types. These proportions constituted the predictor variable. All teeth samples were assigned a binary outcome of either 0 or 1. For each bite mark, the teeth sample originating from the same participant (corresponding) was assigned 1 (to indicate an expected match) and the remaining teeth samples (non-corresponding) assigned 0 (to indicate expected non-matches). To determine whether a relationship existed between the binary outcome variable and the predictor variable, the data from each locus were fitted to individual models. The corresponding p-values indicated whether the binary outcome variable was influenced by the measured predictor variable, thus a p-value less than 0.05 indicated a relationship.

To determine the optimum proportion of shared identical reads yielding the greatest probability of correctly matching a bite mark to the corresponding teeth, the model for each locus was used to estimate values for four different parameters: sensitivity, specificity, positive predictive value (PPV) and negative predictive value (NPV). These parameters assessed the ability of the predictor variable to correctly match a bite mark to the corresponding teeth.

Sensitivity is defined as the proportion of true positives correctly identified as such and specificity is the proportion of correctly identified true negatives [47]. In this study, sensitivity is the proportion of correct bite mark and corresponding teeth matches; specificity is the proportion of correct bite mark and non-corresponding teeth matches. To estimate sensitivity and specificity, each teeth sample had to be classified definitively using a “gold-standard” assessment, in addition to being classified according to the test being assessed. The “gold-standard” assessment was the previously described binary outcome variable. The test being assessed was the ability of the predictor variable to correctly match a bite mark to the corresponding teeth; thus the second assignment of binary values depended on the proportion of shared identical reads between the two sample types. A value of 1 was given if the proportion was higher than the optimum proportion of shared identical reads yielding the greatest probability of correctly matching a bite mark to the teeth responsible. To determine this optimum proportion, a Receiver Operator Characteristic (ROC) analysis was performed. The ROC analysis assessed the performance of different proportions (ranging from the lowest to the highest proportions observed in comparative analyses) to estimate values for sensitivity, specificity, PPV and NPV.

PPV is the proportion of test positives that are truly positive and NPV is the proportion of test negatives that are truly negative [47]. In this study, PPV is the proportion of bite mark and corresponding teeth matches (being assigned a “match” according to the predictor variable) that were correct. NPV is the proportion of bite mark and non-corresponding teeth matches (being assigned a “match” according to the predictor variable) that were correct. Wald confidence intervals were calculated for sensitivity, specificity, PPV and NPV to indicate the range of values for each parameter that are possible 95% of the time under repeated sampling.

## Results and Discussion

### Quality Filtering of Read Data

**Figure 1:**
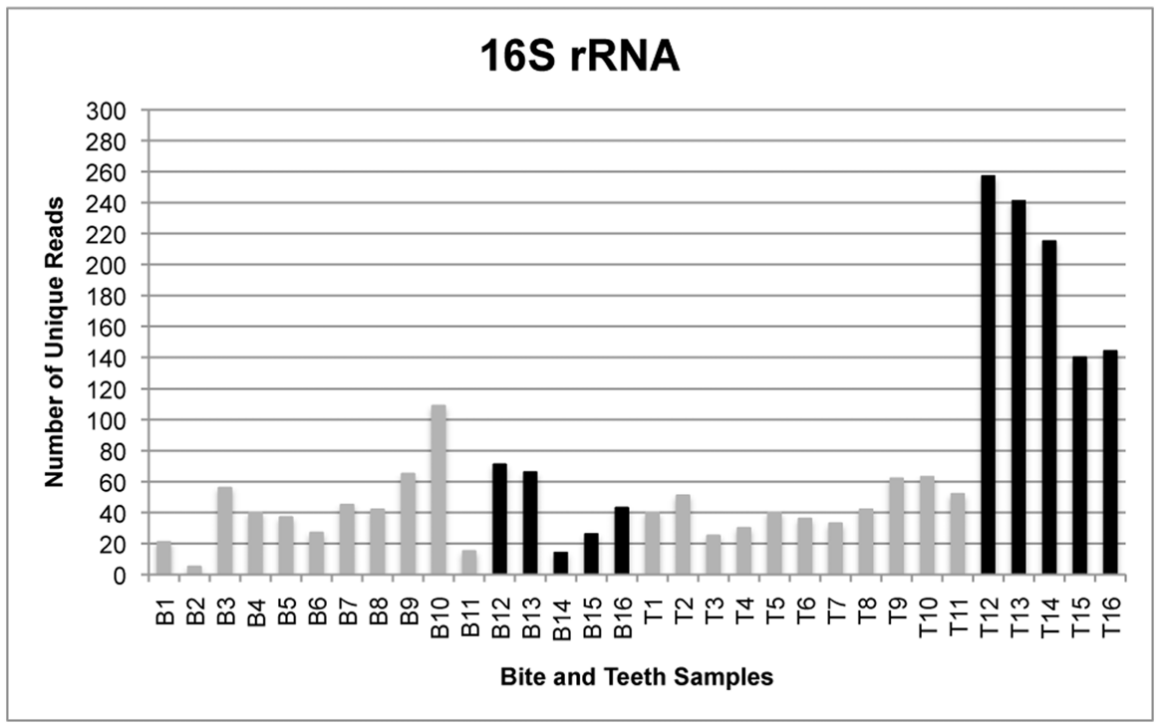
The number of unique reads for single- and multi-locus sequencing of 16S rRNA. Comparison of the number of unique 16S rRNA reads generated from samples in which amplicons from four loci were pooled (gray) and those submitted for sequencing singly (black). Bite mark sample 2 contains less than 10 unique reads and was therefore excluded from comparative analyses for all loci.

**Figure 2:**
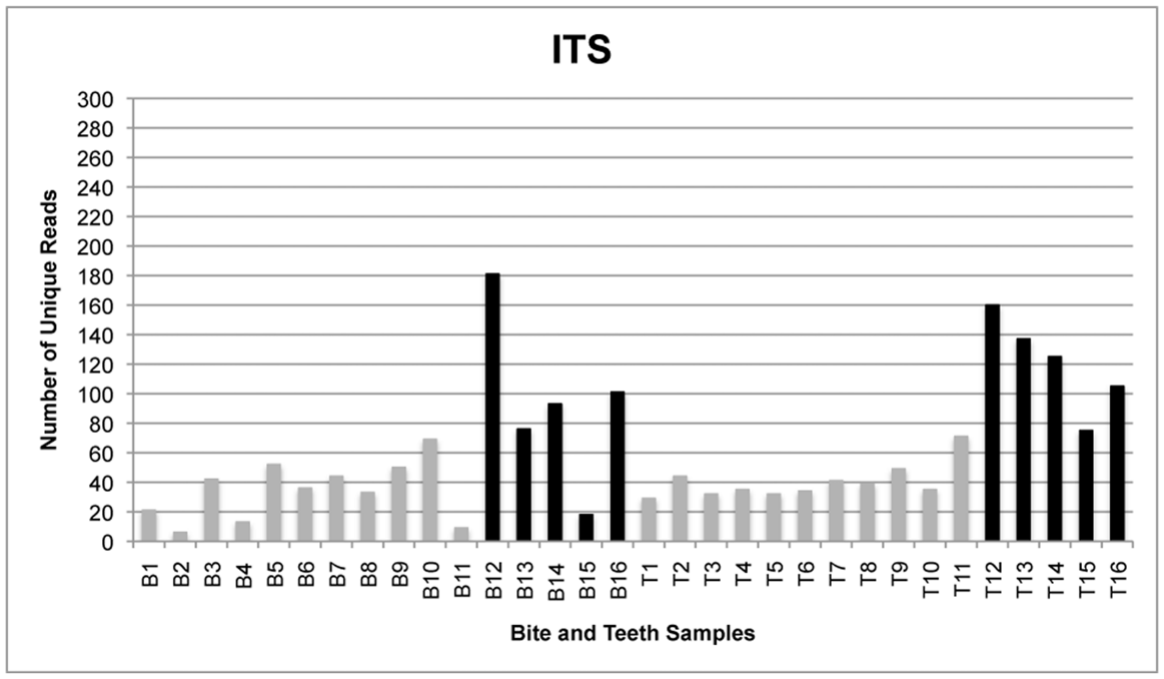
The number of unique reads for single- and multi-locus sequencing of ITS. Comparison of the number of unique ITS reads generated from samples in which amplicons from four loci were pooled (gray) and those submitted for sequencing singly (black). As with 16S rRNA, bite mark sample 2 contains less than 10 unique reads and was therefore excluded from comparative analyses for all loci.

**Figure 3:**
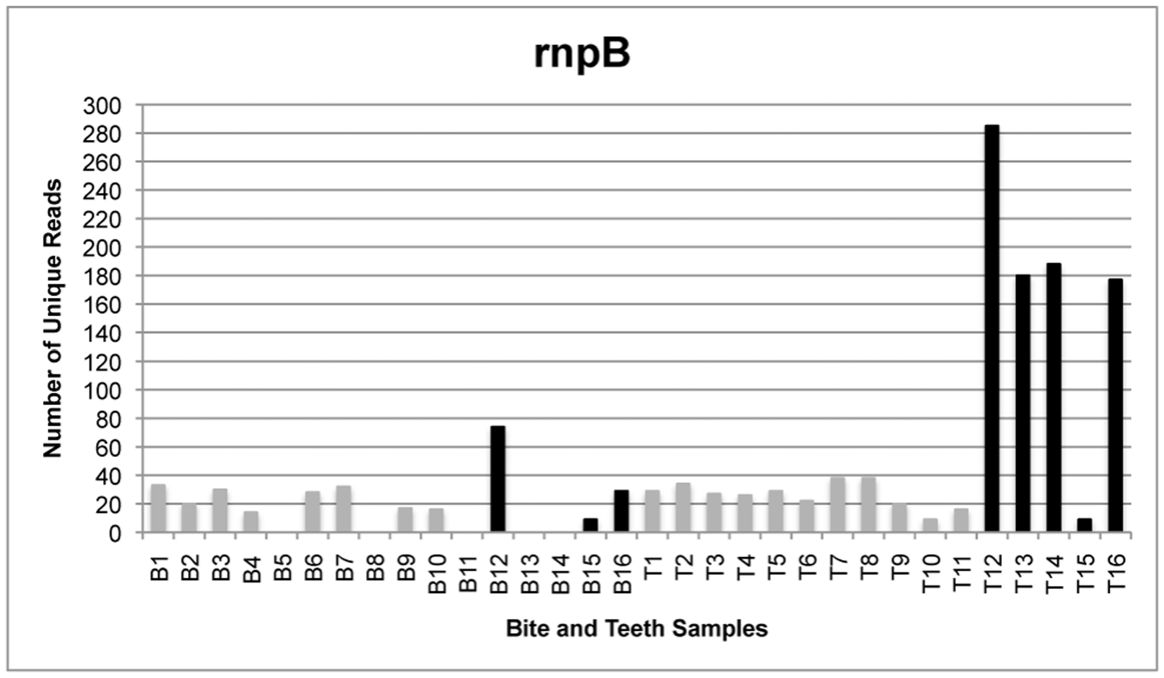
The number of unique reads for single- and multi-locus sequencing of *rnpB*. Comparison of the number of unique *rnpB* reads generated from samples in which amplicons from four loci were pooled (gray) and those submitted for sequencing singly (black). All *rnpB* reads were excluded from comparative analyses because bite mark samples 5, 8, 11, 13, 14 contained no reads following quality filtering and bite mark samples 15 and teeth samples 10 and 15 contained less than 10 unique reads.

**Figure 4:**
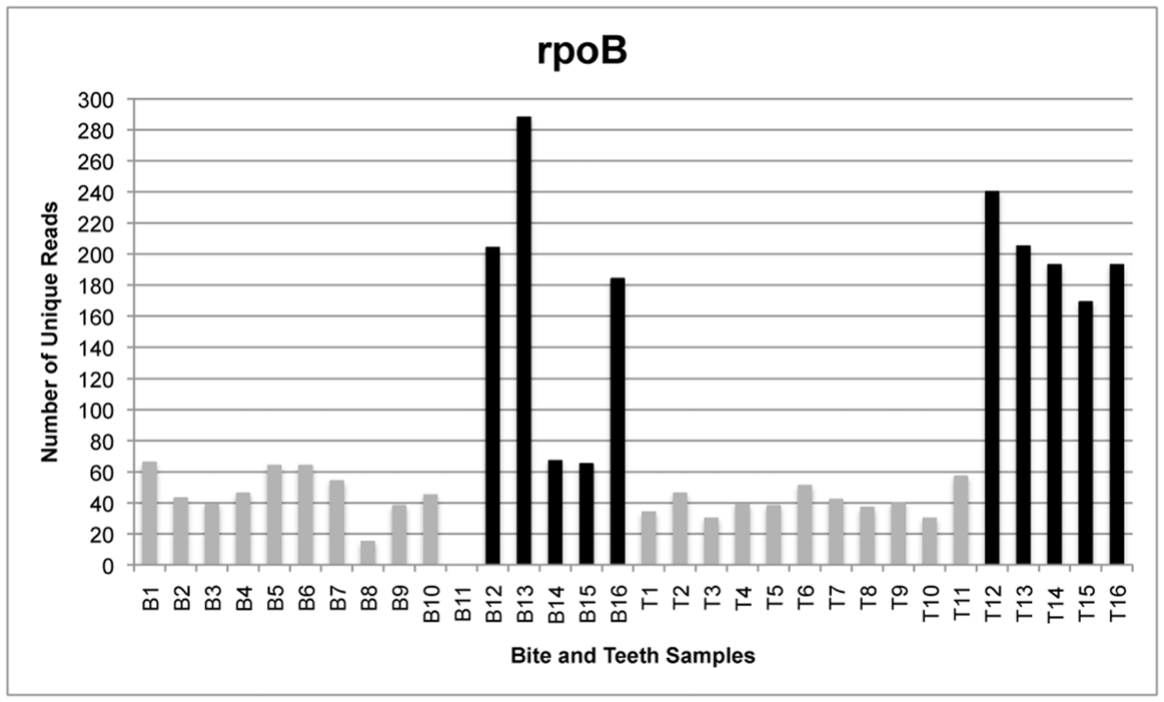
The number of unique reads for single- and multi-locus sequencing of *rpoB*. Comparison of the number of unique *rpoB* reads generated from samples in which amplicons from four loci were pooled (gray) and those submitted for sequencing singly (black). Bite mark sample 11 contains less than 10 unique reads and was therefore excluded from comparative analyses for all loci.

The total number of reads generated by the GS FLX sequencing instrument was 179,987 from all bite mark samples and 232,229 from all teeth samples, translating to 115,801 and 117,886 unique reads for bite mark and teeth samples, respectively. Following quality filtering, the total number of unique reads was 3,164 from all bite mark samples and 5,085 from all teeth samples (Figures 1–4). The average length of sequence reads was 200 bp. This was expected for these amplicons following primer sequence removal.

**Figure 5:**
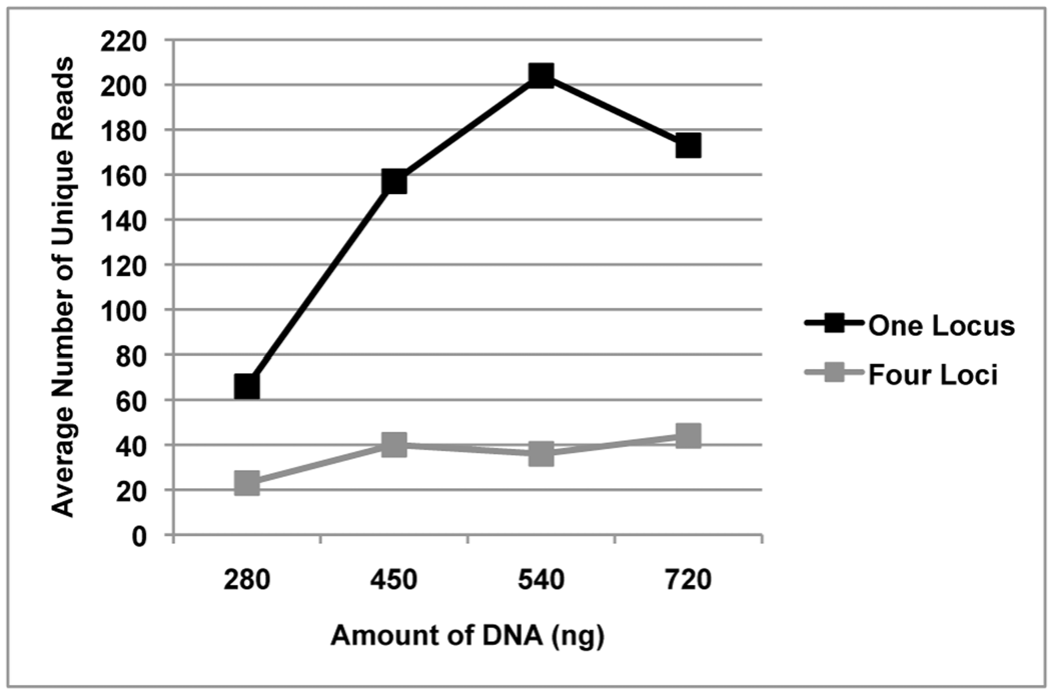
The effect of single- and multi-locus sequencing on unique read numbers. Comparison of the average number of unique reads generated from samples with varying amounts of DNA, containing either amplicons from one locus or amplicons from four loci.

The amplicon libraries from the four loci generated from bite mark and teeth samples 1–11 were pooled prior to sequencing. To determine whether single amplicon sequencing enhanced the number of unique reads, five additional bite mark and teeth samples (B/T12-16) were collected and the amplicons sequenced singly (rather than combined as a pool). Under these conditions, the number of unique reads (remaining after filtering) was generally greater than from samples in which the loci were pooled (Figure 5). Furthermore, submitting higher amounts of DNA for sequencing also increased the average number of unique reads.

**Figure 6:**
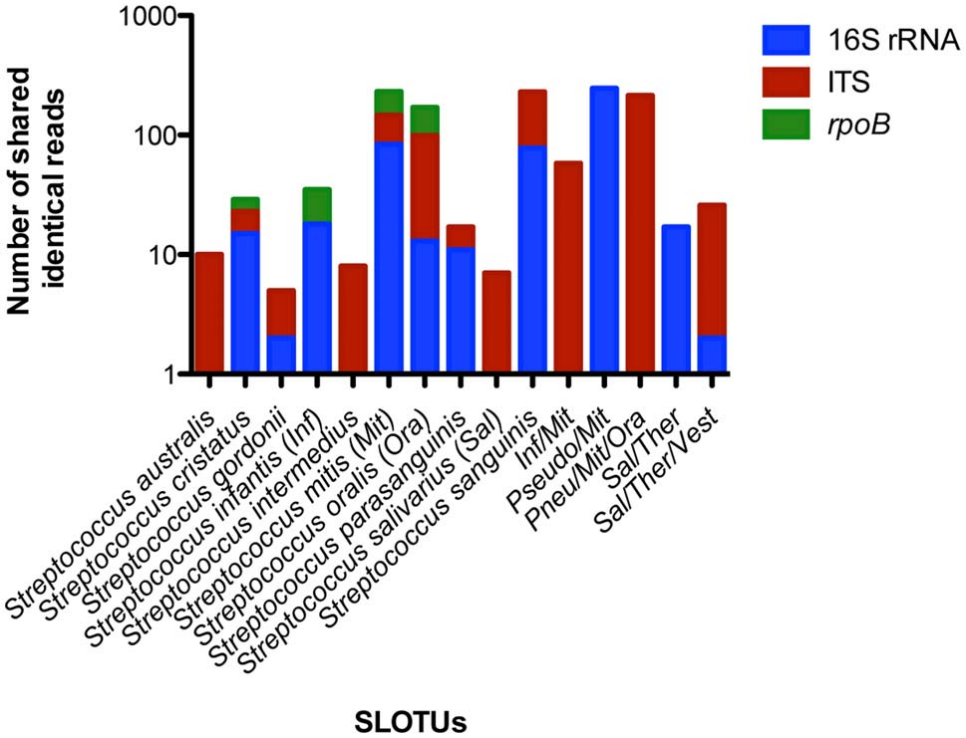
Phylogenetic analysis of identical reads shared between bite mark and teeth samples. All 16S rRNA, ITS and *rpoB* reads identical between bite mark and teeth samples were compiled into locus-specific files and uploaded into GenBank for standard nucleotide-nucleotide BLAST comparison to determine SLOTUs. The number of shared identical reads in each locus-specific file was 482, 639 and 178, respectively. (Pseudo-*Streptococcus pseudopneumoniae*; Pneu-*Streptococcus pneumoniae*; Ther-*Streptococcus thermophilus*; Vest-*Streptococcus vestibularis*).

None of the skin control samples obtained prior to biting generated detectable amplicons using the streptococcus-specific fusion primers designed in this study. Molecular approaches have identified pyogenes group streptococci (e.g. *S. pyogenes*) and oral streptococci from various skin sites using universal bacterial primers for the 16S rRNA gene [48], [49], [50], [51], [52]. However, the specificity of customized primers designed specifically from oral streptococci sequences used in the current study (evidenced by the absence of amplicons from the skin controls) provides assurance that the streptococci amplified from the bite marks originated from the teeth. High stringency filtering of the data to retain only reads that are 100% identical between bite mark and teeth samples further ensured analysis of strictly oral streptococci. This latter measure was validated by performing a phylogenetic analysis of 16S rRNA, ITS and *rpoB* reads that were matched between the two sample types. All shared reads were confirmed as representing species of oral streptococci with *S. mitis*, *S. oralis* and *S. cristatus* being identified by all three loci. The variability within the 16S rRNA and ITS regions were insufficient for distinguishing between closely related oral streptococci within the mitis and salivarius groups (Figure 6).

### Comparison of Bite Mark and Teeth Read Data

Tables 2, 3 and 4 compare the proportions of identical 16S rRNA, ITS and *rpoB* reads shared between bite mark and teeth samples. After filtering, each retained sample contained at least ten unique reads. Samples 2 and 11 were excluded as they contained less than ten unique reads. *RnpB* reads were also excluded from comparative analyses because most samples contained less than ten unique reads following filtering (Figure 3).

For pooled samples, a comparison of 16S rRNA, ITS and *rpoB* reads revealed that the highest proportion of identical reads occurred between bite mark and corresponding teeth samples in 8, 7 and 9 (of 9) comparisons, respectively (Tables 2–4). For individually sequenced samples (12–16), a comparison of 16S rRNA, ITS and *rpoB* reads revealed that the highest proportion of identical reads occurred between bite mark and corresponding teeth samples in 5 (of 5) comparisons, for each locus (Tables 2–4). A comparison of the unique reads from the teeth samples of all participants revealed that on average, 11% of 16S rRNA reads and 20% of ITS reads were common to all participants. In contrast, participants shared only 1.6% of *rpoB* reads.

To determine whether the greater number of 16S rRNA and ITS unique reads obtained by single amplicon sequencing improved the discriminatory capabilities of these regions, read data from pooled samples (1–11) were compared with the read data from the singly sequenced samples (12–16). The increased number of unique 16S rRNA reads from teeth samples 13 and 15 produced proportions of identical reads with bite mark samples 3, 5, 6 and 9 that were greater than those obtained with their corresponding teeth samples (Table 2). The increased number of unique ITS reads from teeth sample 16 produced proportions of identical reads with bite mark samples 9 and 15 that were greater than that from teeth sample 16 (Table 3). In contrast, the increased number of unique *rpoB* reads obtained from teeth samples 12–16 did not produce proportions with bite mark samples 1–11 that exceeded those obtained with their corresponding teeth samples (Table 4).

**Table 2:** Proportions of unique identical 16S rRNA reads shared between bite mark and teeth samples included in comparative analyses.

	B1	B3	B4	B5	B6	B7	B8	B9	B10	B12	B13	B14	B15	B16
**T1**	0.29	0.13	0.10	0.14	0.07	0.13	0.07	0.09	0.07	0.08	0.11	0.03	0.15	0.14
**T3**	0.14	0.20	0.05	0.19	0.11	0.09	0.12	0.09	0.05	0.06	0.06	0.01	0.04	0.16
**T4**	0.05	0.05	**0.28**	0.08	0.15	0.04	0.05	0.06	0.05	0.03	0.05	0.01	0.08	0.05
**T5**	0.14	0.09	0.08	0.38	0.30	0.09	0.12	0.14	0.08	0.04	0.12	0.02	0.15	0.09
**T6**	0.19	0.13	0.05	0.16	0.30	0.16	0.17	0.14	0.08	0.01	0.02	0	0	0
**T7**	0.14	0.05	0.03	0.19	0.19	**0.27**	0.07	0.11	0.06	0.03	0.11	0.01	0.15	0.07
**T8**	0.05	0.11	0.10	0.03	0.07	0	**0.31**	0.09	0.06	0.03	0.06	0.01	0.04	0.05
**T9**	0.19	0.16	0.08	0.19	0.15	0.13	0.17	0.38	0.09	0.01	0.09	0	0.12	0.09
**T10**	**0.33**	0.14	0.05	0.19	0.26	0.13	0.17	0.11	**0.36**	0.06	0.09	0.03	0.04	0.07
**T12**	0.19	0.20	0.10	0.16	0.15	0.07	0.26	0.18	0.06	**0.35**	0.18	0.05	0.12	0.26
**T13**	0.33	**0.30**	0.18	**0.43**	0.30	0.20	0.29	**0.40**	0.17	0.20	**0.52**	0.06	0.19	0.26
**T14**	0.24	0.16	0.10	0.30	0.15	0.09	0.19	0.17	0.10	0.10	0.14	**0.21**	0.12	0.12
**T15**	0.29	0.25	0.10	0.41	**0.37**	0.18	0.29	0.14	0.14	0.15	0.23	0.07	**0.31**	0.16
**T16**	0.33	0.21	0.15	0.30	0.33	0.16	0.31	0.23	0.14	0.20	0.27	0.08	0.19	**0.40**

Bold numbers indicate the highest proportion of identical reads in each column. Sample 2 contained less than 10 unique reads therefore was omitted from comparative analyses. Bite mark samples (B) and teeth samples (T) from the same participant have corresponding identifying numbers. The number of *rpoB* reads in bite mark sample 11 was less than 10 therefore was omitted from comparative analyses of all loci. For bite mark sample 1, teeth samples 10, 13 and 16 share the same proportion however, teeth sample 10 was selected because this data set contained the least number of reads (Figure 1).

**Table 3:** Proportions of unique identical ITS reads shared between bite mark and teeth samples included in comparative analyses.

	B1	B3	B4	B5	B6	B7	B8	B9	B10	B12	B13	B14	B15	B16
**T1**	**0.52**	0.19	0.38	0.17	0.11	0.23	0.27	0.16	0.17	0.07	0.13	0.10	0.28	0.12
**T3**	0.38	**0.36**	0.54	0.23	0.17	0.25	0.24	0.20	0.12	0.07	0.16	0.08	0.22	0.11
**T4**	0.33	0.19	**0.69**	0.17	**0.28**	0.14	0.18	0.16	0.09	0.05	0.13	0.05	0.17	0.08
**T5**	0.24	0.17	0.31	**0.35**	0.19	0.18	0.15	0.18	0.10	0.07	0.14	0.05	0.17	0.10
**T6**	0.19	0.10	0.23	0.15	0.25	0.07	0.09	0.10	0.07	0.05	0.13	0.08	0.11	0.06
**T7**	0.33	0.19	0.38	0.23	0.11	**0.39**	0.27	0.12	0.16	0.04	0.13	0.04	0.11	0.06
**T8**	0.19	0.07	0.15	0.10	0.06	0.18	0.48	0.10	0.16	0.09	0.13	0.03	0.06	0.11
**T9**	0.29	0.19	0.38	0.21	0.11	0.18	0.21	0.30	0.14	0.03	0.08	0.03	0.11	0.04
**T10**	0.33	0.14	0.38	0.13	0.08	0.25	**0.48**	0.18	**0.33**	0.09	0.21	0.04	0.06	0.06
**T12**	0.48	0.29	0.46	0.35	0.25	0.34	0.27	0.38	0.22	**0.24**	0.33	0.06	0.17	0.18
**T13**	0.52	0.33	0.46	0.35	0.22	0.30	0.24	0.32	0.25	0.18	**0.45**	0.12	0.33	0.23
**T14**	0.29	0.17	0.23	0.31	0.17	0.25	0.21	0.28	0.13	0.08	0.14	**0.32**	0.11	0.12
**T15**	0.24	0.21	0.23	0.19	0.22	0.23	0.18	0.34	0.13	0.12	0.18	0.03	**0.28**	0.20
**T16**	0.43	0.33	0.54	0.21	0.19	0.27	0.24	**0.40**	0.19	0.14	0.25	0.10	0.39	**0.29**

Bold numbers indicate the highest proportion of identical reads in each column. Bite mark samples (B) and teeth samples (T) from the same participant have corresponding identifying numbers. The number of 16S rRNA reads in bite mark sample 2 and the number of *rpoB* reads in bite mark sample 11 was less than 10 therefore were omitted from comparative analyses of all loci. For bite mark sample 8, the corresponding teeth sample and teeth sample 10 share the same proportion however, the latter was selected because this data set contained the least number of reads (Figure 2).

**Table 4:** Proportions of unique identical *rpoB* reads shared between bite mark and teeth samples included in comparative analyses.

	B1	B3	B4	B5	B6	B7	B8	B9	B10	B12	B13	B14	B15	B16
**T1**	**0.21**	0	0	0.02	0.03	0.04	0	0	0.07	0	0.01	0	0.02	0.02
**T3**	0	**0.41**	0	0.02	0.02	0.02	0.13	0	0	0.03	0	0.03	0.02	0.01
**T4**	0	0.03	**0.37**	0.06	0	0	0	0	0	0	0	0	0	0
**T5**	0	0	0.04	**0.31**	0.02	0	0	0	0.02	0	0	0.04	0.02	0.01
**T6**	0	0.03	0	0	**0.27**	0	0.07	0.05	0	0.01	0.02	0.09	0.02	0.03
**T7**	0.02	0	0	0.02	0.02	**0.20**	0.07	0	0.07	0	0	0.03	0.02	0.01
**T8**	0.02	0.03	0	0	0.02	0.02	**0.27**	0	0	0	0	0	0	0.01
**T9**	0	0	0.02	0	0.03	0	0	**0.39**	0.02	0.01	0	0.01	0	0.03
**T10**	0.03	0.08	0	0.02	0.05	0	0	0	**0.38**	0.01	0.01	0.01	0.02	0.01
**T12**	0	0	0	0	0	0.02	0	0	0.04	**0.27**	0	0.03	0.05	0.01
**T13**	0	0	0.04	0.03	0.03	0	0.07	0.03	0.02	0	**0.22**	0.01	0.06	0.02
**T14**	0	0	0.09	0.03	0	0	0	0.08	0.02	0.01	0.03	**0.16**	0.02	0.05
**T15**	0	0.03	0.04	0.05	0	0.02	0.07	0	0	0.02	0.02	0.15	**0.15**	0.01
**T16**	0	0.08	0.02	0.02	0.02	0	0	0.16	0.02	0.01	0.04	0.06	0.03	**0.16**

Bold numbers indicate the highest proportion of identical reads in each column. Bite mark samples (B) and teeth samples (T) from the same participant have corresponding identifying numbers. Bite mark sample 11 contained less than 10 unique reads therefore was omitted from comparative analyses of all loci. The number of 16S rRNA reads in bite mark sample 2 was less than 10 therefore was omitted from comparative analyses of all loci.

### Statistical Analysis

Pooled sequence data (i.e. samples 1 and 3–10) were fitted to logistic regression models as the change in methodology disqualified samples 12–16. Table 5 lists the statistical parameters determined by logistic regression modelling.

**Table 5:** The effectiveness of each diagnostic model (i.e. locus) for predicting the probability of obtaining a correct match between a bite mark and the teeth responsible.

Model	p	OP	Sens.(%)	Spec.(%)	PPV(%)	NPV(%)	AUC
16S rRNA	<0.001	0.2	100	96	75	100	0.99
			(55–100)	(88–99)	(43–95)	(92–100)	
ITS	<0.001	0.25	100	76	35	100	0.92
			(55–100)	(65–86)	(17–56)	(90–100)	
rpoB	<0.001	0.2	100	100	100	100	1.0
			(55–100)	(93–100)	(55–100)	(93–100)	

Probabilities (p) determined that the assignment of a binary outcome variable was influenced by the proportion of shared identical reads between bite and teeth samples; OP- optimum proportion of shared identical reads yielding the greatest estimates of sensitivity, specificity, PPV and NPV; Sens- Sensitivity; Spec- Specificity; PPV- positive predictive values; NPV- negative predictive value. Values in the brackets indicate the lower and upper values of a 95% confidence interval.

Tables 2, 3 and 4 indicate that in at least 7 (of 9) comparisons, the highest proportion of shared identical reads occurred between a bite mark and its corresponding teeth sample. This strongly suggests that matching a bite mark to the teeth responsible is dependent on the predictor variable (i.e. proportion of shared identical reads). The probabilities confirm that the binary outcome variable was influenced by the measured predictor variable (Table 5) and not by some unmeasured variable or chance.

Assessment of the ability of the predictor variable to correctly match a bite mark to the corresponding teeth was provided by model estimates for specificity, sensitivity, PPV and NPV. ROC analysis revealed the optimum proportion of shared identical reads yielding the greatest values for each of the four parameters (Table 5). For the 16S rRNA model, the sensitivity of 100% indicates that all bite marks will be matched to the corresponding teeth; however, the PPV predicts that the proportion of these matches being correct is 75% (i.e. 25% false positive rate). The occurrence of false positives was also observed in the previously reported method involving the analysis of 16S rRNA amplicon profiles resolved by DGGE [22]. For the ITS model, a maximized sensitivity yielded a PPV of 35%, translating to a 65% chance of obtaining a false positive. The values for the *rpoB* model revealed maximized scores of 100% for all four diagnostic measures indicating that all bite marks will be correctly matched to the corresponding teeth. Furthermore, the *16S rRNA*, ITS and *rpoB* models all exhibit maximum negative predictive values, assuring that all negative cases will be correctly assigned (Table 5).

Under repeated random sampling from the population, the confidence intervals indicate the boundaries that will contain the true value of each parameter 95% of the time. It is important to recognize that bite mark evidence attempts to confirm the identity of a person held on suspicion based on other evidence. In other words, the approach explored here is not aimed at identifying an assailant from the wider population in the absence of other indicative evidence. Also derived from the ROC analyses are the values for the area under the curve (AUC), which measures the overall ability to discriminate between samples from teeth responsible for a bite and those not responsible, when compared to any bite mark sample. Where perfect discrimination is attained the ROC curve yields an area of 100%. The strength of the *rpoB* model was reiterated with an AUC of 100% (Table 5).

Fitting a model to sample data primarily involves finding estimates of the model parameters that are in some sense “optimal” for the data. Confidence that the estimates derived from each model are optimal was established by calculating two parameters, pseudo R^2^ and goodness of fit, which assessed the appropriateness of each model (data not shown). The pseudo R^2^ was calculated to indicate the proportion of variability in the data that is explained by the model. For 16S rRNA and ITS, the models explained 71% and 34% of the variability, respectively. The pseudo R^2^ of 100% obtained for *rpoB*, revealed a model that explains all of the variability in the data, thus constituting the best model. The “goodness of fit” tests the null hypothesis that the model approximates the data; a value of ≥ 0.05 is required for the model to be deemed a good fit of the data. While the 16S rRNA and ITS models met this criterion with values of 0.3 and 0.08 respectively, the *rpoB* model was exceedingly strong with a value of 0.996.

Of the three loci assessed, *rpoB* was clearly the most satisfactory, providing unequivocal identification of the teeth responsible for each bite. The strength of this region was validated in three ways: firstly, the high stringency of the filtering process ensured that data sets contained reads of the highest quality; thus correctly matching a bite with the teeth responsible was achieved using 3% of the initial unique reads. Secondly, the average proportion of identical reads shared between bite marks and corresponding teeth samples was an order of magnitude greater than those of bite mark and non-corresponding teeth samples. This ratio was maintained when the original bite samples were compared with teeth samples 12–16, which were sequenced at greater depth. Thirdly, the predictive power of *rpoB* to correctly assign a bite mark to the teeth responsible was absolute and supported by both AUC and PPV.

The differing performances of the three regions in distinguishing between participants can be attributed to the target sites of each primer. The 16S rRNA and ITS primers amplify a range of streptococcal species whereas the *rpoB* primers were designed to amplify only *S. mitis*, the most prevalent species on tooth surfaces [30], [31], [32], [33], [34]. The robustness of *rpoB* in distinguishing participants is due to exclusivity to a species with profound genotypic diversity therefore permitting coverage of that species at a greater depth. The variable regions enclosed within the 16S rRNA and ITS fragments do not offer the discriminatory power required to distinguish between participants as irrefutably as does the *rpoB* region.

From a forensic standpoint, assurance that there is temporal stability of oral streptococcal populations is crucial. Genetic analyses reveal that oral streptococcal populations are dynamic with species numbers and proportions fluctuating over time [34], [53]. The mechanisms underlying these changes are not fully understood; however, the dominant strains of streptococci are generally retained over longer periods [21], [34], [53]. Approximately 20% of all *S. mitis* genotypes recovered from the buccal mucosae of six participants were detected in repeated samplings over a 10-month period [34], and almost 50% of *S. mitis* and *S. oralis* genotypes from two individuals were detected two years after initial sampling [53]. Rahimi *et al.,* [21] found that between 20–78% of bacterial genotypes were recovered from the same teeth 12 months later. Nevertheless, the likelihood of matching bite mark sequence data to that of a suspected assailant will be increased by prompt sampling.

In conclusion, the comparison of highly discriminatory regions of oral streptococcal DNA recovered from bite marks and teeth is capable of unequivocally matching a bite mark to the teeth responsible and may provide valuable information to corroborate other evidence in cases where the perpetrators DNA cannot be recovered.
